# Symptoms of depression and anxiety increased marginally from before to during the COVID-19 pandemic among young adults in Canada

**DOI:** 10.1038/s41598-022-20379-1

**Published:** 2022-09-26

**Authors:** Marie-Pierre Sylvestre, Gillis D. Tchouangue Dinkou, Alexia Armasu, Annie Pelekanakis, Vickie Plourde, Mathieu Bélanger, Katerina Maximova, Brett D. Thombs, Jennifer O’Loughlin

**Affiliations:** 1 Centre de recherche du centre hospitalier de l’Université de Montréal (CRCHUM), 850 Saint-Denis (S03-458), Montreal, QC H2X 0A9 Canada; 2 Department of Social and Preventive Medicine, Université de Montréal, Montreal, QC Canada; 3 School of Psychology, Université de Moncton, Moncton, NB Canada; 4 Department of Family Medicine, Université de Sherbrooke, Sherbrooke, QC Canada; 5 Centre de Formation Médicale du Nouveau-Brunswick, Université de Sherbrooke, Moncton, NB Canada; 6 Vitalité Health Network, Moncton, NB Canada; 7 MAP Centre for Urban Health Solutions, LiKa Shing Knowledge Institute, St. Michael’s Hospital, Toronto, ON Canada; 8 Dalla Lana School of Public Health, University of Toronto, Toronto, ON Canada; 9 Faculty of Medicine, McGill University, Montreal, QC Canada; 10 Lady Davis Institute for Medical Research, Jewish General Hospital, Montreal, QC Canada

**Keywords:** Anxiety, Depression, Risk factors

## Abstract

We documented changes in depressive and anxiety symptoms from before to during the COVID-19 pandemic among young adults and investigated whether changes differed across participant characteristics. Data were available in an investigation of 1294 grade 7 students recruited in 1999–2000. For this analysis, we used data collected pre-pandemically in 2017–20 (mean (SD) age = 30.6 (1.0)) and during the pandemic in 2020–21 (mean (SD) age = 33.6 (0.6)). 673 participants with data in both cycles were retained for analysis. Symptoms were measured using the Major Depression Inventory (MDI) and the Generalized Anxiety Disorder-7 (GAD-7) scale. Standardized mean differences (SMD) of changes in MDI and GAD-7 values between cycles were calculated across participant characteristics. On average, MDI scores increased by 2.1 (95%CI 1.4, 2.8) points during the pandemic from mean 10.5; GAD-7 scores increased by 1.2 (0.8, 1.5) points from mean 4.7. The SMD was 0.24 (0.14, 0.33) for MDI, and 0.24 (0.13, 0.34) for GAD-7. No differences in MDI change scores were observed across participant characteristics. Differences in GAD-7 change scores were observed by mood/anxiety disorder (SMD − 0.31 (− 0.58, − 0.05)), household income (0.24 (0.02, 48)), living with young children (− 0.56 (− 1.23,− 0.05)), and adherence to public health recommendations 0.58 (0.19, 1.03)). Increases in depressive and anxiety symptoms were observed 10–16 months into the COVID-19 pandemic among adults age 32–36.

## Introduction

There is growing evidence that the COVID-19 pandemic and associated containment measures impacted mental health negatively, especially early in the pandemic^1,2^ when isolation, social network disruptions, precarious employment situations, and limited access to usual health care were intensified^3–5^. Evidence from past, smaller-scale pandemics^6–10^ suggests that the negative impact could persist post-pandemic^11–15^. However, changes in mental health could also reflect an acute response to the pandemic that eventually wanes so that mental health returns to pre-pandemic levels. A systematic review of 65 longitudinal studies reported that increases in depressive and anxiety symptoms early in the pandemic (March–April 2020) were modest, although greater than those reported later (May–July 2020)^16^. Further, while small increases compared to pre-pandemic levels remained apparent in depressive symptoms, anxiety symptom levels were close to pre-pandemic levels^16^. However, this review had several limitations, including high attrition in most studies included^16^. In addition, it reported high heterogeneity in mental health across samples that was not explained by country-specific characteristics (i.e., number of COVID-19 cases and/or attributed deaths^16^), supporting the need for local studies to inform the Canadian context.

Studies of individual change in mental health in Canada, which to date are limited to 6-months post-pandemic onset, report mixed findings, including no deterioration in mental health among high school students^17^ or 22 year-old adults^18^ and increased anxiety and depressive symptoms in mothers age 25–54 years^19^. Findings from a large, population-based representative sample in the UK with repeated measures of mental health before and in the early months of the pandemic, suggested that the 25–34 age group was among the hardest hit with higher increases in mental distress noted among young adults with children age 0–5, women and the unemployed^20^. In addition to the effects of lockdown, disruption of education, loss of income, unemployment, anxiety about contracting COVID-19, and ever-changing public-health recommendations may have caused accumulating feelings of uncertainty about the future in young adults. Thus, studying mental health symptoms across stages of the pandemic is critical to understanding whether symptoms persist. In addition, from a developmental perspective, young adults are in a highly transitional stage in the life course during which they establish long-term relationships, raise children, establish careers, buy homes and build financial security^21,22^. Given 75% of mental health disorders have a first onset before age 25^23^, empirical evidence highlighting vulnerability to the effects of COVID-19 among young adults with history of mental health disorders is warranted.

The objectives of this study were to describe changes in depressive symptoms and anxiety levels from before to 10–16 months into the COVID-19 pandemic in adults age 32–36, and to assess whether the magnitude of change differed across subgroups defined by history of a diagnosis of a mood or anxiety disorder, sociodemographic characteristics, and self-reported adherence to public health recommendations.

## Methods

### Data source

Data were drawn from the Nicotine Dependence in Teens (NDIT) study^24^, an ongoing prospective study that initially enrolled 1294 participants age 12–13 recruited in 7th grade from 10 Montreal-area high schools in 1999–2000. A purposive sample of schools was selected to ensure a mix of schools located in rural, suburban and urban neighbourhoods, schools with French or English-speaking students, and schools serving advantaged and disadvantaged populations. Student characteristics at cycle 1 were comparable to those reported in the 1999 provincially representative Québec Child and Adolescent Health and Social Survey^24,25^.

The current study used post-high school data collected in cycle 23 when participants were age 30.6 years on average (SD = 1.0) and in cycle 24 (mean age 33.6 (0.6)). Data from cycle 23 were collected between January 2017 and March 12, 2020, the day before a health emergency was declared in Québec. Data from cycle 24 were collected during the COVID-19 pandemic from December 2020 to June 2021 (i.e., 10–16 months after declaration of the COVID-19 public health emergency in Québec). Following the second wave of COVID-19 during Fall 2020, a 5-month curfew was implemented in Québec beginning January 2021^26^. During data collection for cycle 24, the stringency index (i.e., a measure of the severity of policies related to containment restrictions with high values representing more stringent policies^27^) decreased from 63.4 to 48.7.

### Measurements

Depressive symptoms were measured using the *Major Depression Inventory (MDI)*, a validated 12-item self-report questionnaire investigating mood in the past two weeks using a 6-point Likert-type response scale^28,29^. *MDI* scores ranged from 0 to 50 with higher scores indicative of more frequent depressive symptoms.

Anxiety severity was measured using the 7-item self-report *Generalized Anxiety Disorder-7 (GAD-7)* scale^30^. *GAD-7* scores range from 0 to 21 with higher scores indicative of more severe symptoms of anxiety.

*History of a mood/anxiety disorder diagnosis* (yes, no) was self-reported in cycle 23 and defined as having been diagnosed by a health professional with a mood (i.e., depression, bipolar disorder) or anxiety (i.e., phobia, fear of social situations, obsessive–compulsive disorder, panic disorder, generalized anxiety disorder) disorder.

Sociodemographic characteristics measured at cycle 24 included *age*, *sex, genetically-determined ancestry* (primarily European, other), *born in Canada* (yes, no), *lives in urban/suburban setting* as indicated by postal codes (yes, no), *lives alone* (yes, no), *lives with children* (yes, no), *highest education* (high school, more than high school), *unemployed* (yes, no)*,* and *household income* (< 50,000$, ≥ 50,000$ CAN).

COVID-19 pandemic-related variables were measured in cycle 24 and included *single-parent in households with children* (yes, no), ≥ *1 child age* ≤ *5 years in households with children* (yes, no), *work-at-home status among the employed* (works at home, works outside home/mixed*),* and three items measuring whether participants worried often in the past 2 weeks (yes, no) about: (i) *being or becoming unemployed*; (ii) *not being able to pay your bills*; and (iii) *not being able to visit people who depend on you*. *Self-reported adherence to public health recommendations* was measured by: ‘Indicate your level of agreement with the following. In general, I follow public health recommendations on COVID-19’. Responses choices were recoded as low if participants responded *Strongly disagree, Disagree* or *Neither agree nor disagree,* and high if participants responded *Agree or Strongly agree.*

Questionnaire items, response choices, recoding of responses, and Cronbach’s α for the *MDI* and *GAD*-7 as well as in independent studies are described in Supplementary Table S1.

### Statistical analysis

Non-response weights to account for attrition in cycle 24 were estimated using inverse-probability weighting. Derivation of the weights is described in Supplementary Material Table S2. Missing values other than in *MDI* or *GAD-7* that were not due to attrition (≤ 5.2% per variable) were not imputed (Supplementary Table S3). Each analysis was based on the maximum number of non-missing values for the corresponding variables. Unless otherwise indicated, results were weighted for non-response.

We considered within-individual change (i.e., Δ) in *MDI* and *GAD-7* scores in all participants with data in both cycles 23 and 24 and according to history of a mood/anxiety disorder diagnosis, sociodemographic characteristics, and COVID-19 pandemic-related variables. We computed standardized mean differences (SMD) to measure mean within individual change in *MDI* and *GAD-7* scores and we investigated differences in changes related to pandemic-related variables measured in cycle 24. For example, SMD for self-reported adherence to public health recommendations was computed as the difference in mean Δ between individuals with high vs. low self-reported adherence to public health recommendations, divided by the within-group standard deviation averaged across cycle 23 and 24^31^. The advantage of an analysis of within-individual change is that individuals act as their own comparator. 95% confidence intervals (CI) for SMD were obtained using bootstrap resampling.

In sensitivity analyses assessing whether time elapsed between completing cycle 23 and 24 questionnaires impacted the results, mean *MDI* and *GAD-7* scores were calculated in samples stratified at the median number of days between cycles. Analyses were performed using R version 4.0.4 (R Foundation for Statistical Computing, Vienna, Austria) with packages *ggeffects*, *Hmisc* and *boot.*

### Ethics

NDIT was approved by ethics committees at the Montreal Department of Public Health, McGill University and the Centre de recherche du Centre hospitalier de l’Université of Montréal, including the amendment for the COVID-19 study (2007–2384, 2017–6895, ND06.087, 2021–9385, 20.278-YP). Parents/guardians provided informed written consent at inception, and participants provided consent post-high school. All methods were performed in accordance with the relevant guidelines and regulations and reported according to the STROBE guidelines (Supplementary Material).

## Results

A total of 713 participants (55.1% of 1294 NDIT participants) responded to cycle 24. A total of 799 participants completed cycle 23 questionnaires; 5 of 799 who completed the cycle 23 questionnaire after the health emergency was declared in Québec on March 13, 2020 were excluded to ensure that cycle 23 captured pre-pandemic measures of mental health only. Supplementary Material Fig. S1 shows the flow chart of response from NDIT baseline to cycle 24. Within-individual analyses were performed using the analytical sample which comprised 673 participants with complete data in cycles 23 and 24. Compared to participants not retained in the analyses (n = 621), a lower proportion of those retained were male, they were more likely to have university-educated mothers and less likely to have tried alcohol or cigarettes in cycle 1 (Supplementary Material Table S4).

Mean (95% CI) *MDI* and *GAD-7* scores were 12.4 (11.7, 13.0) and 5.8 (5.5, 6.1), respectively 10–16 months into the pandemic (Table 1 for *MDI;* Table 2 for *GAD-7*), reflecting increases over pre-pandemic levels of $\overline{\Delta  } =$ 2.1 (1.4, 2.8) points for *MDI* (SMD 0.24 (0.14, 0.33)) and $\overline{\Delta  } =$ 1.2 (0.8, 1.5) points for *GAD-7* scores (SMD 0.24 (0.13, 0.34)), respectively. Results stratified by time between cycles were similar (Supplementary Material Tables S5–S8).

**Table 1:** Weighted^a^ cycle-specific means, mean of within-individual differences (Δ) and standardized mean changes (SMD) for *MDI* scores from cycle 23 to 24, Nicotine Dependence in Teens study, Québec, Canada 2017–2021, n = 673.

Characteristics	Cycle specific values		Within-individual differences from cycle 23 to 24	
	Mean cycle 23 (95% CI)	Mean cycle 24 (95% CI)	Mean Δ^b^ (95% CI)	SMD^c^ (95% CI)
All	10.3 (9.6, 10.9)	12.4 (11.7, 13.1)	2.1 (1.4, 2.8)	**0.24 (0.14, 0.33)**
**History of a mood/anxiety disorder diagnosis**				− 0.14 (− 0.43, 0.14)
No	8.7 (8.0, 9.4)	11.2 (10.4, 11.9)	2.4 (1.6, 3.2)	
Yes	14.9 (13.7, 16.2)	16.2 (14.9, 175)	1.2 (− 0.1, 2.6)	
**Sex**				0.11 (− 0.09, 0.29)
Male	9.0 (8.0, 10.1)	10.6 (9.6, 11.7)	1.6 (0.5, 2.6)	
Female	11.1 (10.3, 11.9)	13.6 (12.7, 14.4)	2.5 (1.6, 3.3)	
**Ancestry**				0.04 (− 0.21, 0.30)
European	9.9 (9.2, 10.7)	12.1 (11.3, 12.8)	2.2 (1.4, 3.0)	
Other	11.6 (10.3, 13.0)	14.2 (12.8, 15.5)	2.5 (1.1, 3.9)	
**Born in Canada**				0.12 (− 0.26, 0.52)
No	11.0 (8.4, 13.6)	12.1 (9.5, 14.8)	1.1 (− 1.6, 3.8)	
Yes	10.2 (9.6, 10.9)	12.4 (11.7, 13.1)	2.2 (1.5, 2.9)	
**Lives in urban/suburban setting**				0.13 (− 0.11, 0.40)
No	10.5 (9.0, 12.0)	11.7 (10.2, 13.3)	1.2 (− 0.4, 2.8)	
Yes	10.3 (9.5, 11.0)	12.6 (11.8, 13.3)	2.3 (1.6, 3.1)	
**Lives alone**				0.21 (− 0.05, 0.52)
No	10.0 (9.4, 10.7)	11.9 (11.2, 12.6)	1.8 (1.1, 2.6)	
Yes	11.5 (9.9 13.1)	15.1 (13.5 16.8)	3.6 (2.0, 5.3)	
**Lives with children**				− 0.06 (− 0.25, 0.14)
No	10.9 (10.0, 11.7)	13.2 (12.3, 14.1)	2.3 (1.4, 3.2)	
Yes	9.6 (8.6, 10.5)	11.4 (10.4, 12.4)	1.9 (0.9, 2.9)	
**Single parent in a household with children**				0.03 (− 1.17, 0.75)
No	9.2 (8.3, 10.2)	11.1 (10.1, 12.1)	1.8 (0.8, 2.9)	
Yes	13.8 (10.4, 17.3)	15.9 (12.4, 19.5)	2.1 (− 1.7, 5.9)	
**Age of children in households with children**				0.02 (− 0.81, 0.56)
≥ 1 child age ≤ 5 years	9.1 (8.2, 10.1)	11.0 (9.9, 12.0)	1.8 (0.8, 2.9)	
Children all age > 5 years	13.6 (10.8, 16.4)	15.6 (12.7, 18.5)	2.0 (− 1.1, 5.2)	
**Highest education**				0.08 (− 0.30, 0.57)
More than high school	10.3 (9.7, 11.0)	12.4 (11.7, 13.1)	2.1 (1.4, 2.8)	
High school	11.7 (7.9, 15.5)	14.5 (10.6, 18.4)	2.8 (− 1.1, 6.8)	
**Unemployed**				0.08 (− 0.19, 0.34)
No	10.0 (9.3, 10.7)	12.0 (11.3, 12.7)	2.0 (1.3, 2.8)	
Yes	11.8 (10.2, 13.3)	14.5 (12.8, 16.1)	2.7 (1.0, 4.3)	
**Household income**				0.08 (− 0.19, 0.34)
< $50,000	12.9 (11.6, 14.2)	14.5 (13.1, 15.8)	1.6 (0.2, 3.0)	
≥ $50,000	9.5 (8.8, 10.2)	11.8 (11.1, 12.6)	2.3 (1.5, 3.1)	
**Work-at-home status during COVID-19 among employed**				− 0.12 (− 0.36, 0.11)
Works at home	11.1 (9.9, 12.2)	13.7 (12.6, 14.9)	2.7 (1.5, 3.8)	
Works outside home/mixed	9.2 (8.4, 10.1)	11.0 (10.1, 11.8)	1.7 (0.8, 2.6)	
**Self-reported adherence to public health recommendations**				0.09 (− 0.22, 0.47)
Low	12.5 (10.7, 14.3)	13.9 (12.1, 15.8)	1.5 (− 0.4, 3.4)	
High	10.0 (9.3, 10.7)	12.2 (11.5, 12.9)	2.2 (1.5, 2.9)	
**Worried about being unemployed**				0.17 (− 0.09, 0.42)
No	9.8 (9.1, 10.4)	11.7 (11.0, 12.4)	1.9 (1.2, 2.7)	
Yes	13.7 (12.0, 15.4)	17.0 (15.2, 18.7)	3.3 (1.5, 5.1)	
**Worried about not being able to pay bills**				0.12 (− 0.19, 0.43)
No	9.6 (8.9, 10.3)	11.6 (10.9, 12.3)	2.0 (1.3, 2.7)	
Yes	14.7 (13.0, 16.4)	17.7 (16.0, 19.5)	3.0 (1.2, 4.8)	
**Worried about not being able to visit people who depend on you**				0.12 (− 0.15, 0.39)
No	9.7 (8.9, 10.4)	11.6 (10.8, 12.4)	1.9 (1.1, 2.7)	
Yes	12.5 (11.2, 13.8)	15.4 (14.0, 16.8)	2.9 (1.5, 4.3)	

^a^Weighted for non-response.

^b^Represents individual differences between MDI or GAD-7 scores in cycle 24 and 23.

^c^Represents differences in mean change between groups, divided by the within-group standard deviation averaged across cycle 23 and 24. 95% CIs were calculated using bootstrap resampling.

SMD with 95% CI that excluded the null are in bold font.

**Table 2:** Weighted^a^ cycle-specific means, mean of within-individual differences (Δ) and standardized mean changes (SMD) for *GAD-7* scores from cycle 23 to 24, Nicotine Dependence in Teens study, Québec, Canada 2017–2021, n = 673.

Characteristics	Cycle specific		Within-individual differences from cycle 23 to 24	
	Mean cycle 23 (95% CI)	Mean cycle 24 (95% CI)	Mean Δ^b^ (95% CI)	SMD^c^ (95% CI)
All	4.7 (4.3, 5.0)	5.8 (5.5, 6.2)	1.2 (0.8, 1.5)	**0.24 (0.13, 0.34)**
**History of a mood/anxiety disorder diagnosis**				**− 0.31 (− 0.58, − 0.05)**
No	3.7 (3.3, 4.1)	5.2 (4.8, 5.6)	1.5 (1.1, 1.9)	
Yes	7.4 (6.7, 8.1)	7.5 (6.8, 8.2)	0.1 (− 0.6, 0.8)	
**Sex**				0.03 (− 0.16, 0.22)
Male	3.6 (3.0, 4.1)	4.7 (4.1, 5.2)	1.1 (0.5, 1.7)	
Female	5.4 (4.9, 5.8)	6.6 (6.1, 7.0)	1.2 (0.7, 1.7)	
**Ancestry**				0.11 (− 0.12, 0.37)
European	4.6 (4.2, 5.1)	5.7 (5.3, 6.1)	1.0 (0.6, 1.5)	
Other	4.8 (4.1, 5.6)	6.4 (5.6, 7.1)	1.6 (0.8, 2.3)	
**Born in Canada**				0.13 (− 0.24, 0.50)
No	4.5 (3.1, 6.0)	5.1 (3.7, 6.5)	0.6 (− 0.9, 2.1)	
Yes	4.7 (4.3, 5.0)	5.9 (5.5, 6.2)	1.2 (0.8, 1.6)	
**Lives in urban/suburban setting**				0.13 (− 0.16, 0.48)
No	4.6 (3.8, 5.5)	5.3 (4.4, 6.1)	0.6 (− 0.2, 1.5)	
Yes	4.7 (4.3, 5.1)	5.9 (5.5, 6.3)	1.2 (0.8, 1.6)	
**Lives alone**				0.04 (− 0.22, 0.29)
No	4.6 (4.2, 5.0)	5.7 (5.3, 6.1)	1.1 (0.7, 1.5)	
Yes	5.1 (4.2, 6.0)	6.4 (5.5, 7.3)	1.3 (0.4, 2.2)	
**Lives with children**				0.03 (− 0.17, 0.23)
No	4.8 (4.3, 5.3)	5.9 (5.4, 6.4)	1.1 (0.6, 1.6)	
Yes	4.4 (3.9, 5.0)	5.6 (5.1, 6.2)	1.2 (0.6, 1.8)	
**Single parent in a household with children**				− 0.29 (− 1.02, 0.39)
No	4.3 (3.7, 4.9)	5.6 (5.1, 6.1)	1.3 (0.7, 1.9)	
Yes	6.3 (4.3, 8.2)	6.2 (4.4, 8.0)	0.0 (− 2.0, 2.0)	
**Age of children in households with children**				**− 0.56 (− 1.23, − 0.05)**
≥ 1 child age ≤ 5 years	4.1 (3.4, 4.7)	5.6 (5.0, 6.1)	1.5 (0.9, 2.1)	
Children all age > 5 years	7.5 (5.9, 9.1)	6.4 (4.8, 7.9)	− 1.1 (− 2.8, 0.5)	
**Highest education**				− 0.37 (− 1.31, 0.08)
More than high school	4.6 (4.2, 5.0)	5.8 (5.4, 6.1)	1.2 (0.8, 1.5)	
High school	6.9 (5.0, 8.9)	6.4 (4.5, 8.2)	− 0.5 (− 2.5, 1.4)	
**Unemployed**				− 0.06 (− 0.43, 0.23)
No	4.4 (4.0, 4.8)	5.6 (5.2, 6.0)	1.2 (0.8, 1.6)	
Yes	5.9 (5.0, 6.8)	6.8 (5.9, 7.7)	0.9 (0.0, 1.8)	
**Household income**				**0.24 (0.02, 0.48)**
< $50,000	5.7 (5.0, 6.5)	6.0 (5.3, 6.8)	0.3 (− 0.5, 1.0)	
≥ $50,000	4.3 (3.9, 4.8)	5.7 (5.3, 6.1)	1.4 (1.0, 1.8)	
**Work-at-home status during COVID-19 among employed**				− 0.23 (− 0.44, 0.00)
Works at home	4.8 (4.2, 5.4)	6.6 (6.0, 7.2)	1.8 (1.2, 2.4)	
Works outside home/mixed	4.2 (3.7, 4.6)	5.0 (4.5, 5.5)	0.8 (0.4, 1.3)	
**Self-reported adherence to public health recommendations**				**0.58 (0.19, 1.03)**
Low	7.4 (6.4, 8.3)	6.2 (5.2, 7.1)	− 1.2 (− 2.2, − 0.2)	
High	4.3 (3.9, 4.6)	5.7 (5.4, 6.1)	1.5 (1.1, 1.9)	
**Worried about being unemployed**				− 0.01 (− 0.30, 0.26)
No	4.3 (3.9, 4.7)	5.4 (5.1, 5.8)	1.1 (0.8, 1.5)	
Yes	7.0 (6.0, 7.9)	8.0 (7.1, 8.9)	1.1 (0.1, 2.1)	
**Worried about not being able to pay bills**				− 0.05 (− 0.35, 0.24)
No	4.3 (3.9, 4.6)	5.4 (5.0, 5.8)	1.2 (0.8, 1.6)	
Yes	7.1 (6.2, 8.1)	8.1 (7.3, 9.0)	0.9 (0.0, 1.9)	
**Worried about not being able to visit people who depend on you**				0.03 (− 0.27, 0.30)
No	4.1 (3.7, 4.5)	5.2 (4.8, 5.6)	1.1 (0.7, 1.5)	
Yes	6.6 (5.8, 7.3)	7.8 (7.1, 8.5)	1.2 (0.5, 2.0)	

^a^Weighted for non-response.

^b^ Represents individual differences between MDI or GAD-7 scores in cycle 24 and 23.

^c^ Represents differences in mean change between groups, divided by the within-group standard deviation averaged across cycle 23 and 24. 95% CIs were calculated using bootstrap resampling.

SMD with 95% CI that excluded the null are in bold font.

Within-individual analyses suggested no important differences in the change in *MDI* between groups defined by history of a mood/anxiety disorder diagnosis, sociodemographic characteristics, and pandemic-related variables (Tables 1 and 2; Supplementary Tables S9 for unweighted results). All 95% CIs for the SMD included the null value. Within-individual changes in *GAD-*7 were less consistent across participant characteristics. Participants without a mood/anxiety diagnosis reported a 1.5-point increase in *GAD-*7 scores, although those with a diagnosis remained stable over time (i.e., SMD comparing the two groups − 0.31 (− 0.58, − 0.05)). Participants with younger children (i.e., age ≤ 5) reported increases in *GAD-7* scores, while those with older children reported declines (i.e., SMD − 0.56 (− 1.23, − 0.05)). Compared to participants with higher incomes, those with incomes ≤ $50,000 reported higher mean *GAD-7* scores both before and during the pandemic. However, the magnitude of increases over time was larger among participants with incomes ≥ $50,000 (i.e., SMD 0.24 (0.02, 0.48)). Finally, high self-reported adherence to public health COVID-19 recommendations was associated with increases in *GAD-7* scores. Low self-reported adherence was associated with decreases in *GAD-7* scores (SMD was 0.58 (0.19, 1.03)).

## Discussion

Our results suggest that depressive and anxiety symptoms were higher than pre-pandemic levels 10–16 months into the COVID-19 pandemic among Canadian adults age 32–34 years. SMDs were higher than those reported in a recent meta-analysis^16^ (i.e., 0.24 (0.14, 0.33) vs. 0.12 (0.14, 0.30) for depressive symptoms; 0.24 (0.13, 0.34) vs. 0.13 (0.02, 0.23) for anxiety symptoms), possibly because of differences in containment measures and policies across countries and/or in the timeline and scales used to measure mental health^16^. The meta-analysis suggested that acute increases in anxiety symptoms early in the pandemic (March–April 2020) waned over the next few months (May–July 2020)^16^, although increases in depressive symptoms remained constant. Our study suggests that pandemic-related impacts on both depressive and anxiety symptoms persisted at least 10–16 months into the pandemic. Four findings are salient.

First, our report that changes in anxiety symptoms were associated with household income aligns with the UK study^20^ conducted early in the pandemic in a large representative sample, but not with the two longitudinal studies in Canadian adults^18,19^. The UK study observed a U-shape relationship between quintiles of equivalized household income and mental health in which the two extreme quintiles were associated with larger decrease in mental health. We report that participants with a higher income had a larger increase in anxiety symptoms than participants with lower household income. Our sample size did not allow for a more precise description of household income. The UK study further reported that unemployed participants reported a decrease in mental health while the employed reported an increase, a finding that was consistent with the *All Our Families* study of Canadian mothers^19^. We did not observe any association between employment status and mental health, which may be due to the timing of the measurement of mental health between studies. The *All Our Families* measured mental health between May and July 2020, during which the employment rate in Canada was slowly increasing after its large drop in April 2020, while our measures were performed after December 2020 when the employment rate was almost back to its pre-pandemic level^32^.

Second, living with younger vs older children was associated with larger changes in anxiety symptoms. This aligns with the UK study^20^ but differs from the *All Our Families* study, which reported no differences in mental health based on the age of children^19^. However, *All Our Families* did observe larger increases in depressive and anxiety symptoms in mothers who reported difficulty balancing home schooling with work responsibilities, or obtaining childcare^19^. Discrepancies across studies could relate in part to differences in the availability^33^ and cost^34^ of childcare in Québec (where most NDIT participants reside), compared to other Canadian provinces, including Alberta, where *All Our Families* was conducted.

Third, change in anxiety scores from before to during the pandemic differed according to a history of a mood/anxiety disorder diagnosis. Participants without a mood/anxiety diagnosis reported an increase in anxiety scores, while those with a diagnosis remained stable over time. This could be explained by a ceiling effect among those with a history of mood/anxiety disorder diagnosis or a catch-up effect in the group without a diagnosis. Alternatively, participants with a history of mood/anxiety disorder diagnosis may have developed coping strategies before the pandemic that helped/prepared them to manage COVID-19-related depressive and anxiety symptoms. Our results differ from the aforementioned meta-analysis^1,16^ and the *All Our Families* study^19^, which did not identify differences in change in mental health related to a history of mental health disorder diagnoses. Discrepancies across studies could reflect differences in definitions and accuracy of self-reported history of diagnoses^35^, and/or in access to mental health services across jurisdictions^36^. Despite burgeoning availability of online resources to address mental distress during COVID-19, disruptions or delays in accessing mental health services may have increased levels of unmet needs during the pandemic and underpin the association observed in our study^37^.

Fourth, a systematic review of 11 longitudinal studies reported gender or sex differences in changes in anxiety and general mental health from before to during the pandemic, but not in stress or depression levels (Dal Santo et al. unpublished manuscript available on medRxiv, 10.1101/2021.06.28.21259384)). Although women in NDIT reported higher levels of depressive and anxiety symptoms than men before and during the pandemic, the magnitude of the change over time did not differ by sex. Women assumed much of the additional burden associated with homeschooling, domestic chores and addressing family well-being early in the pandemic^38,39^, but this higher burden may not have translated into differential changes in mental health. Alternatively, women already had or they developed resilience to face these new challenges (Dal Santo et al. unpublished manuscript available on medRxiv, 10.1101/2021.06.28.21259384)).

Investigations are needed to assess whether the impact of the pandemic on mental health persists as new variants emerge, containment measures change, and pandemic-related economic impacts such as inflation endure^40^. Further, mechanisms underpinning impacts on mental health need to be identified^41^. Longitudinal studies with multiple observations during the pandemic will help us to move beyond simple ‘before-after’ studies that are prone to regression to the mean^42^. Qualitative studies on coping mechanisms and resilience may help increase understanding of heterogeneity in mental health responses to the pandemic. Studies are needed that focus on racialized/marginalized populations^43^ and growing pandemic-related inequalities in mental health^44^. For example, studies must move beyond sex differences to investigate changes in mental health in gender and sexual minorities. These groups in addition to racialized individuals, were already subject to important health inequities before the pandemic, and are more likely to report poorer perceived mental health^45,46^.

### Strengths and limitations

Strengths of this study include use of validated measures of depressive and anxiety symptoms, and that we accounted for time elapsed between measurements^47,48^. Limitations include possible selection bias related to attrition. Use of non-response weights likely attenuated the impact of attrition, although the validity of the weights depends on the missing-at-random assumption, which was not testable in the data. While our analysis was constrained by the size of the existing cohort, the width of the 95% confidence intervals for our estimates of change in mental health and corresponding standardized mean differences suggests that the precision of our estimates is reasonable. The descriptive nature of our analysis precludes causal interpretations. Because 77% of participants were of European ancestry, our ability to investigate racialized groups was limited. Changes in anxiety and depressive symptoms between cycles 23 to 24 may not be entirely attributable to COVID-19 and/or may capture regression to the mean^42^. Findings may not generalize to other jurisdictions or age groups.

## Conclusion

Depressive and anxiety symptoms were elevated over pre-pandemic levels 10–16 months into the pandemic in our study of adults age 32–36 years. Future studies will need to assess whether and when mental health symptoms return to pre-pandemic levels and in addition, identify modifiable mechanisms underpinning any persisting impact of COVID-19 on depressive and anxiety symptoms.

## Supplementary Information

Supplementary Information 1.

Supplementary Information 2.

## Data Availability

NDIT data are available upon request. Access to NDIT data is open to any university-appointed or affiliated investigator upon successful completion of the application process. Masters, doctoral and postdoctoral students may apply through their primary supervisor. To gain access, applicants must complete a data access form available on our NDIT website (http://www.CELPHIE.ca) and return it to the principal investigator (jennifer.oloughlin@umontreal.ca). The procedure to obtain access to NDIT data is described in O'Loughlin et al.^25^.
