# Supplementary material for: Symptoms of depression and anxiety increased marginally from before to during the COVID-19 pandemic among young adults in Canada
Source: Sci Rep. 2022 Sep 26;12:16033. doi: 10.1038/s41598-022-20379-1 (PMC9512816; doi:10.1038/s41598-022-20379-1)
Supplement: Supplementary file 1 — Supplementary Information 1. [file 41598_2022_20379_MOESM1_ESM.docx]

**Supplementary Material**

Table S1. Description of questionnaire item(s), response options and recoding for analysis for study variables, Nicotine Dependence in Teens study, Québec, Canada, 1999-2021

Table S2. Derivation and distribution of non-response weights, Nicotine Dependence in Teens study, Québec, Canada, 2007-2021

Table S3. Number of missing values among participants at cycles 23 and 24, Nicotine Dependence in Teens study, Montreal, Canada 2017-2021

Table S4. Characteristics of participants at study inception (1999-2000) according to retainment status in the analytical sample, Nicotine Dependence in Teens Study, Québec, Canada, 1999-2021

Figure S1. Flow chart describing the derivation of the analytical samples, Nicotine Dependence in Teens study, Montreal, Canada, 1999-2021.

Table S5. Weighted means and differences for change in MDI and GAD-7 scores from cycle 23 to 24, time below the median, Nicotine Dependence in Teens study, Montreal, Canada 2017-2021

Table S6. Weighted means and differences for change in MDI and GAD-7 scores from cycle 23 to 24, time above the median, Nicotine Dependence in Teens study, Montreal, Canada 2017-2021

Table S7. Unweighted means and differences for change in MDI and GAD-7 scores from cycle 23 to 24, time below the median, Nicotine Dependence in Teens study, Montreal, Canada 2017-2021

Table S8. Unweighted means and differences for change in MDI and GAD-7 scores from cycle 23 to 24, time above the median, Nicotine Dependence in Teens study, Montreal, Canada 2017-2021

Table S9. Unweighted mean of individual differences (Δ) and standardized mean changes (SMΔ) for MDI and GAD-7 scores from cycle 23 to 24, Nicotine Dependence in Teens study, Montreal, Canada 2017-2021, n=673

**Table S1. Description of questionnaire item(s), response options and recoding for analysis for study variables, Nicotine Dependence in Teens Study, Québec, Canada, 1999-2021**

| **Variable (reference)** | **Data drawn from cycle(s)** | **Item(s)** | **Response choices** | **Recoding for analysis** |
| --- | --- | --- | --- | --- |
| **Sociodemographic indicators** | | | | |
| Age | 1-24 | Cycle 1-24: What is today’s date?  Cycle 1-20: In what month is your birthday?  Cycle 1-20: On what day of the month is your birthday?  Cycle 1-20: In what year were you born? | (day) (month) (year) | Used to compute age in years |
| Sex | 1-23 | Are you a boy or a girl? | Male; Female |  |
| Ancestry | Genetic data of 962 NDIT participants | European ethnicity was assessed using data on DNA | NA | Yes (primarily European);  No (other) |
| Born in Canada | 1 | Were you born…? | In Canada; outside Canada | Yes (In Canada);  No (outside Canada) |
| Lives in urban/suburban setting^1^ | 24 | Participant's population centre, calculated according to their postal code and the Postal CodeOM Conversion File (PCCF), Statistics Canada, December 2017 | Unknown; Rural area; Small population centre (1,000 to 29,999); Medium population centre (30,000 to 99,999); Large urban population centre (100,000 or greater) | Yes (if lives in an Urban area);  No (if lives in a Rural area) |
| Lives alone | 21-24 | Do you currently live alone? | No; Yes | No;  Yes |
| Lives with children | 24 | Are there children living with you at your current place of residence?  What is your current marital status? | No; Yes  Single; Married; Common-law; Divorced; Separated; Other (specify) | No;  Yes, as single parent (if they were single, divorced or separated);  Yes, with another parent (if they were married or in common-law) |
| Number of children at home | 24 | What is the age of the youngest child? | Age of each child (6 entries possible) | None (if lives with children = no);  ≥ 1 child age ≤ 5 years;  All children age > 5 years |
| Highest education | 21-24 | How far have you gone in school? | Attended high school, but did not graduate; Graduated high school; Attended CEGEP, community/technical college, but did not graduate; Graduated CEGEP, community/technical college; Attended university (or teacher’s college), but did not graduate; Graduated university with a Bachelor’s degree; Graduated university with a Master’s degree; Graduated university with a PhD/(professional degree); Other (specify) | High school;  More than high school;  NA |
| Unemployed | 21-24 | Are you currently working at a job or business (paid or unpaid)? | No; Yes | Yes (if unemployed);  No (if employed) |
| Household income | 21-24 | What is your best estimate of the total income, before taxes and deductions, of all household members from all sources in the past 12 months? | <20,000$; 20,000-29,999$; 30,000-39,999$; 40,000-49,999$; 50,000-59,999$; 60,000-69,999$; 70,000-79,999$; 80,000-99,999$; 100,000-119,999$; 120,000-149,999$; ≥150,000$; Don’t know | <$50,000;  ≥$50,000 |
| **History of mental health** | | | | |
| History of mood/anxiety disorder diagnosis | 21-23 | Has a health professional ever diagnosed that you have any of the following?  (i) mood disorder (depression, bipolar disorder)  (ii) anxiety disorder (phobia, fear of social situations, obsessive-compulsive disorder, panic disorder, generalized anxiety disorder) | No; Yes | No  Yes |
| **Variables for inverse-probability weighing** | | | | |
| Speaks French at home | 1 | What language do you speak most often at home? | English; French; English and French; Other | Yes (if French; English and French);  No (if English; Other) |
| Mother attended university | 9 and  mother’s questionnaire | Indicator of the highest level of education the mother attained; | Did not graduate high school; High school graduate; Vocational, technical school (even if not graduated); University (even if not graduated); Other; Don’t know; Not applicable | Yes (if University);  No (if Did not graduate high school, High school graduate, Vocational, technical school, CEGEP, Other, Don’t know, Not applicable) |
| Lives in a single parent family | 1 | Check the box if you live with the person. If you live in more than one household, check all the boxes that apply.  i) biological mother  ii) biological father  iii) step-mother  iv) step-father  v) aunt(s)  vi) uncle(s)  vii) grandmother(s)  viii) grandfather(s)  x) other(s) | Yes; No | Yes (if only one adult is endorsed);  No (if otherwise) |
| Kandel depressive symptoms score^2^ | 1 | During the past 3 months, how often have you …?  i) Felt too tired  ii) Had trouble going to sleep  iii) Felt unhappy, sad  iv) Felt hopeless about the future  v) Felt nervous or tense  vi) Worried too much about things | Never; Rarely; Sometimes; Often | Sum of 6 items (1 to 4) from Kandel scale divided by number of items responded to, to create a continuous score (range 1 to 4) |
| Ever smoked a cigarette | 1 | Have you ever in your life smoked a cigarette, even just a puff (drag, hit, haul)? | No; Yes, 1 or 2 times; Yes, 3 or 4 times; Yes, 5 to 10 times; Yes, more than 10 times | No;  Yes (if otherwise) |
| Used alcohol in past 3 months | 1 | During the past 3 months, how often did you… drink alcohol (beer,…)? | Never; A bit to try; Once or a couple of times a month; Once or a couple of times a week; Usually everyday | No (Never);  Yes (if otherwise) |
| **COVID-19** | | | | |
| Work-at-home status among the employed | 24 | Do you go to your workplace in person? | No, I work from home; Mixed (go to workplace and work from home); Yes, I go to my workplace in person; I prefer not to answer | Works at home (if No)  Works outside home/mixed (if Mixed or Yes) |
| Self-reported adherence to public health recommendations | 24 | Indicate your level of agreement with the following. In general, I follow public health recommendations on COVID-19. | Strongly disagree; Disagree; Neither agree nor disagree; Agree; Strongly agree | Low (if Strongly disagree, Disagree or Neither agree nor disagree)  High (if Agree or Strongly agree) |
| Worry in the past 2 weeks | 24 | In the past 2 weeks, how often did you worry about…:  (i) being or becoming unemployed  (ii) not being able to pay your bills  (iii) not being able to visit people who depend on you. | Never; Rarely; Some of the time; Often; Very often | Yes (if Rarely; Some of the time; Often; Very often)  No (if Never) |
| **Mental health** | | | | |
| Depression score (MDI)^3-4^ | 21-24 | In the past two weeks, how much of the time have you…?  (i) felt low in spirits or sad  (ii) lost interest in, or could no longer enjoy your daily activities  (iii) felt lacking in energy and strength  (iv) felt less self-confident  (v) had a bad conscience or feelings of guilt  (vi) felt that life wasn’t worth living  (vii) had difficulty concentrating (when reading the newspaper or watching TV),  (viii) felt very restless  (ix) felt subdued or slowed down  (x) had trouble sleeping at night or waking up too early  (xi) suffered from reduced appetite  (xii) suffered from increased appetite | At no time; Some of the time; Slightly less than half of the time; Slightly more than half of the time; Most of the time; All the time | Responses to each item were summed to create a continuous score (range: 0-50). For items (viii) and (ix), the highest score was retained for scoring. Similarly, for items (xi) and (xii), the highest score was retained for scoring. Cronbach’s α was 0.898 and 0.892 in cycles 23 and 24, respectively.  Excellent internal consistency has been reported in independent studies (Cronbach α ranged 0.90-0.94 across studies). Correlation with another depression scale (HAM-D_17_) (*r*=0.86) indicates good external validity. |
| Anxiety score (GAD-7)^5-7^ | 23-24 | In the past 2 weeks, how often have you been bothered by …?  (i) Feeling nervous, anxious, or on edge  (ii) Not being able to stop or control worrying  (iii) Worrying too much about different things  (iv) Trouble relaxing  (v) Being so restless that it’s hard to sit still  (vi) Becoming easily annoyed or irritable  (vii) Feeling afraid as if something awful might happen | Not at all; Several days; Over half of the days; Nearly every day | Responses to each item were summed to create a continuous score (range: 0-21). Cronbach’s α was 0.905 and 0.876 in cycles 23 and 24, respectively.  Excellent internal consistency (Cronbach α=0.92) has been reported in independent studies. Strong psychometric properties are also supported by good test-retest reliability (intraclass correlation=0.83), good criterion, construct, factorial and procedural validity, and correlations with 2 other anxiety scales (i.e., Beck Anxiety Inventory (*r*=0.72) and the Symptom Checklist-90 anxiety subscale (*r*=0.74)) indicating good convergent validity. |

**Reference for Table S1**

1. Postal CodeOM Conversion File (PCCF), 2017. Statistics Canada Catalogue no. 92-154-X.
2. Kandel DB, Davies M. Epidemiology of depressive mood in adolescents: an empirical study. Arch Gen Psychiatry. 1982;39(10):1205–1212.
3. Bech P, Rasmussen NA, Olsen LR, Noerholm V, Abildgaard W. The sensitivity and specificity of the Major Depression Inventory, using the Present State Examination as the index of diagnostic validity. J Affect Disord. 2001 Oct;66(2-3):159-64. doi: 10.1016/s0165-0327(00)00309-8.
4. Bech P, Timmerby N, Martiny K, Lunde M, Soendergaard S. Psychometric evaluation of the Major Depression Inventory (MDI) as depression severity scale using the LEAD (Longitudinal Expert Assessment of All Data) as index of validity. BMC Psychiatry. 2015 Aug 5;15:190. doi: 10.1186/s12888-015-0529-3.
5. Spitzer RL, Kroenke K, Williams JB, Löwe B. A brief measure for assessing generalized anxiety disorder: the GAD-7. Arch Intern Med. 2006 May 22;166(10):1092-7. doi: 10.1001/archinte.166.10.1092.
6. Olsen LR, Jensen DV, Noerholm V, Martiny K, Bech P. The internal and external validity of the Major Depression Inventory in measuring severity of depressive states. Psychol Med. 2003 Feb;33(2):351-6. doi: 10.1017/s0033291702006724. PMID: 12622314.
7. Spitzer RL, Kroenke K, Williams JB, Löwe B. A brief measure for assessing generalized anxiety disorder: the GAD-7. Arch Intern Med. 2006 May 22;166(10):1092-7. doi: 10.1001/archinte.166.10.1092.

**Table S2. Derivation and distribution of non-response weights, Nicotine Dependence in Teens study, Québec, Canada, 2007-2021**

| We used logistic regression models to derive the non-response weights at cycle 24. We regressed an indicator of response (*yes, no*) at cycle 24 on participants’ characteristics measured at NDIT inception, including Kandel depressive symptoms score, sociodemographic characteristics (i.e., sex, age, speaks French at home (yes, no), born in Canada (yes, no), single-parent family (yes, no), and mother attended university (yes, no)), and available indicators of substance use (ever smoked a cigarette (yes, no) and used alcohol in past three months (yes, no)). We considered interactions between sex and the other covariates, but dropped them as they did not improve model fit. We used squared terms to assess the non-linearity of associations between continuous covariates and the indicator of response on the log-odds scale. The Hosmer-Lemeshow goodness of fit test suggested adequate fit (p-value 0.33). We truncated weights at the 5% and 95% percentiles.^1^ The mean and standard deviation of weights for cycle 24 were 2.4 and 1.7. |
| --- |

**Reference for Table S3**

1. Xiao Y, Moodie EEM, Abrahamowicz M. Comparison of Approaches to Weight Truncation for Marginal Structural Cox Models. *Epidemiologic Methods* 2013; **2**(1): 1-20.

**Table S3. Number of missing values among participants at cycles 23 and 24, Nicotine** **Dependence in Teens study, Montreal, Canada 2017-2021**

| Characteristics | Cycle 23  n = 795 | Cycle 24  n=713 | Cycle 23 and 24  n=673 |
| --- | --- | --- | --- |
| History of a mood/anxiety disorder diagnosis | 0 (0) | 0 (0) | 0 (0) |
| Sex | 0 (0) | 0 (0) | 0 (0) |
| Ancestry | 51 (6.4) | 40 (5.6) | 35 (5.2) |
| Born in Canada | 0 (0) | 0 (0) | 0 (0) |
| Lives in urban/suburban setting | 127 (16.0) | 6 (0.8) | 6 (0.9) |
| Lives alone | 8 (1.0) | 3 (0.4) | 3 (0.5) |
| Lives with children | 0 (0) | 3 (0.4) | 0 (0) |
| Number of children at home | NA | 0 (0) | 0 (0) |
| Highest education | 18 (2.3) | 26 (3.7) | 22 (3.3) |
| Unemployed | 7 (0.9) | 3 (0.4) | 3 (0.5) |
| Household income, CAN | 6 (0.8) | 4 (0.6) | 4 (0.6) |
| Work-at-home status during COVID-19 among the employed | NA | 0 (0) | 0 (0) |
| Self-reported adherence to public health recommendations | NA | 1 (0.1) | 1 (0.2) |
| Worried about being unemployed | NA | 1 (0.1) | 1 (0.2) |
| Worried about not being able to pay bills | NA | 1 (0.1) | 1 (0.2) |
| Worried about not being able to visit people who depend on you | NA | 1 (0.1) | 1 (0.2) |

**Table S4. Characteristics of participants at study inception (1999-2000) according to retainment status in the analytical sample, Nicotine Dependence in Teens Study, Québec, Canada, 1999-2021**

|  | **Retained in the analytical sample** | |
| --- | --- | --- |
|  | **Yes**  **(n=673)** | **No**  **(n=621)** |
| Male, % | 41.5 | 55.5 |
| Age (years), mean (SD) | 12.6 (0.2) | 12.9 (0.4) |
| Speaks French at home, % | 29.4 | 30.8 |
| Born in Canada, % | 94.4 | 89.7 |
| Mother attended university, %  No  Yes  NA | 50.4  41.0  8.6 | 34.0  24.5  41.5 |
| Lives in a single-parent family, % | 9.5 | 10.2 |
| Ever smoked a cigarette, % | 26.8 | 39.5 |
| Depression score, mean (SD)^1^ | 2.1 (0.4) | 2.1 (0.4) |
| Used alcohol in past 3 months, % | 41.0 | 47.6 |

^1^Kandel DB, Davies M. Epidemiology of Depressive Mood in Adolescents: An Empirical Study. Arch Gen Psychiatry. 1982;39(10):1205–1212

**Figure S1. Flow chart describing the derivation of the analytical samples, Nicotine Dependence in Teens study, Montreal, Canada, 1999-2021.**


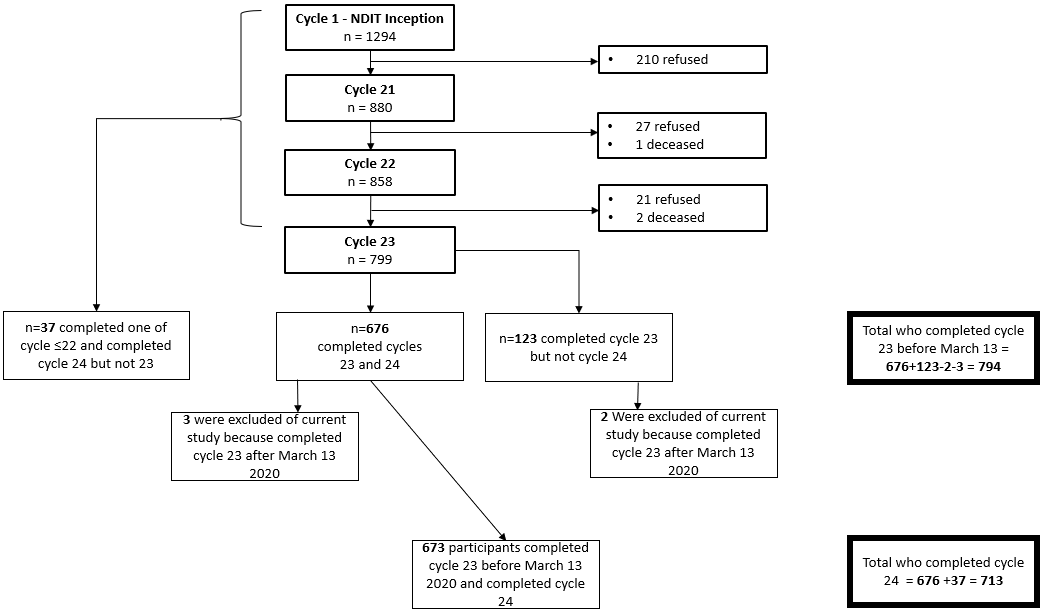


**Table S5. Weighted means and differences for change in MDI and GAD-7 scores from cycle 23 to 24, time below the median, Nicotine Dependence in Teens study, Montreal, Canada 2017-2021 (n=343)**

|  | *MDI†* | | | *GAD-7*^†^ | | |
| --- | --- | --- | --- | --- | --- | --- |
| Characteristics at cycle 24 | Mean cycle 23  (95% CI) | Mean cycle 24  (95% CI) | Mean Δ  (95% CI) | Mean cycle 23  (95% CI) | Mean cycle 24  (95% CI) | Mean Δ  (95% CI) |
| All | 10.5 (9.6, 11.4) | 12.7 (11.8, 13.6) | 2.2 (1.2, 3.2) | 4.7 (4.2, 5.2) | 5.7 (5.2, 6.2) | 1.0 (0.5, 1.5) |
| History of a mood/anxiety disorder diagnosis  No  Yes | 9.2 (8.2, 10.2)  14.7 (12.9, 16.4) | 11.6 (10.5, 12.6)  16.2 (14.3, 18.0) | 2.4 (1.3, 3.5)  1.5 (-0.5, 3.5) | 4.1 (3.6, 4.7)  6.6 (5.7, 7.6) | 5.4 (4.9, 5.9)  6.7 (5.7, 7.6) | 1.3 (0.7, 1.9)  0.1 (-1.0, 1.1) |
| Sex  Male  Female | 9.5 (8.2, 11.0)  11.4 (10.0, 12.3) | 11.4 (10.0, 12.8)  13.6 (12.4, 14.8) | 1.8 (0.3, 3.4)  2.4 (1.2, 3.7) | 3.9 (3.1, 4.6)  5.4 (4.7, 6.0) | 4.8 (4.3, 5.4)  6.5 (6.0, 6.9) | 0.9 (0.1, 1.7)  1.1 (0.4, 1.7) |
| Ancestry  European  Other | 10.3 (9.2, 10.7)  11.0 (10.0, 12.6) | 12.3 (11.2, 13.4)  14.2 (12.3, 16.0) | 2.0 (0.8, 3.1)  3.1 (1.2, 5.1) | 4.9 (4.3, 5.5)  4.5 (3.5, 5.4) | 5.6 (5.0, 6.2)  6.0 (5.1, 6.9) | 0.7 (0.1, 1.3)  1.5 (0.5, 2.6) |
| Born in Canada  No  Yes | 11.6 (8.2, 13.4)  10.4 (9.6, 10.9) | 12.4 (8.8, 16.0)  12.7 (11.8, 13.7) | 0.8 (-3.0, 4.5)  2.3 (1.3, 3.3) | 3.7 (1.8, 5.7)  4.8 (4.3, 5.3) | 4.8 (3.0, 6.7)  5.8 (5.3, 6.3) | 1.1 (-1.0, 3.1)  1.0 (0.4, 1.5) |
| Lives in urban/suburban setting  No  Yes | 11.0 (9.8, 14.8)  10.4 (9.4, 13.8) | 12.3 (9.8, 14.8)  12.8 (11.8, 13.8) | 1.3 (-1.4, 3.9  2.4 (1.3, 3.4) | 5.4 (4.0, 6.7)  4.7 (4.1, 5.2) | 5.6 (4.3, 6.9)  5.7 (5.2, 6.2) | 0.2 (-1.2, 1.6)  1.1 (0.5, 1.6) |
| Lives alone  No  Yes | 10.0 (9.0, 11.0)  12.3 (10.3, 14.3) | 11.8 (10.8, 12.8)  16.4 (14.3, 18.4) | 1.8 (0.7, 2.8)  4.1 (1.9, 6.3) | 4.6 (4.1, 5.2)  5.3 (4.1, 6.4) | 5.5 (4.9, 6.0)  6.6 (5.5, 7.6) | 0.8 (0.3, 1.4)  1.3 (0.2, 2.5) |
| Lives with children  No  Yes | 11.1 (10.0, 12.2)  9.4 (7.9, 10.9) | 13.6 (12.4, 14.7)  11.1 (9.5, 12.6) | 2.5 (1.4, 3.7)  1.7 (0.1, 3.3) | 4.9 (4.3, 5.5)  4.5 (3.7, 5.4) | 5.7 (5.2, 6.3)  5.6 (4.8, 6.4) | 0.9 (0.2, 1.5)  1.0 (0.2, 1.9) |
| Single parent in a household with children  No  Yes | 9.2 (7.9, 10.5)  11.8 (7.1, 16.5) | 10.8 (9.4, 12.3)  14.1 (9.0, 19.2) | 1.7 (0.1, 3.2)  2.3 (-3.3, 7.9) | 4.4 (3.5, 5.2)  6.5 (3.5, 9.5) | 5.6 (4.8, 6.4)  5.4 (2.6, 8.2) | 1.2 (0.3, 2.1)  -1.1 (-4.2, 1.9) |
| Age of children in households with children  ≥ 1 child age ≤ 5 years  Children all age > 5 years | 9.2 (7.8, 10.5)  11.1 (7.1, 15.2) | 10.6 (9.1, 12.0)  15.4 (11.0, 19.7) | 1.4 (-0.2, 3.0)  4.2 (-0.6, 9.0) | 4.4 (3.6, 5.3)  5.6 (2.9, 8.2) | 5.4 (6.4, 6.2)  6.6 (4.2, 9.1) | 1.0 (0.1, 1.9)  1.1 (-1.6, 3.8) |
| Highest education  More than high school  High school | 10.6 (9.6, 11.5)  12.5 (5.3, 19.6) | 12.7 (11.7, 13.7)  17.5 (10.1, 24.9) | 2.2 (1.2, 3.2)  5.0 (-2.8, 12.8) | 4.8 (4.2, 5.3)  7.3 (3.9, 10.8) | 5.7 (5.2, 6.2)  7.7 (4.4, 11.0) | 0.9 (0.4, 1.4)  0.4 (-3.2, 4.0) |
| Unemployed  No  Yes | 10.3 (9.3, 11.3)  11.7 (9.3, 14.1) | 12.3 (11.3, 13.2)  15.4 (13.0, 17.9) | 2.0 (0.9, 3.0)  3.8 (1.2, 6.4) | 4.4 (3.8, 4.9)  7.1 (5.8, 8.4) | 5.5 (5.0, 6.0)  7.0 (5.7, 8.2) | 1.1 (0.6, 1.7)  -0.1 (-1.5, 1.2) |
| Household income  < $50,000  ≥ $50,000 | 12.0 (10.2, 13.8)  10.0 (9.0, 11.0) | 15.5 (13.7, 17.4)  11.8 (10.8, 12.8) | 3.5 (1.6, 5.5)  1.8 (0.7, 2.9) | 5.2 (4.2, 6.9)  4.6 (5.1, 6.1) | 5.9 (5.0, 6.9)  5.6 (5.1, 6.1) | 0.7 (-0.3, 1.8)  1.0 (0.4, 1.6) |
| Work-at-home status during COVID-19 among employed  Works at home  Works outside home/mixed | 11.3 (10.1, 12.6)  9.7 (8.5, 10.9) | 14.4 (13.1, 15.7)  11.1 (9.9, 12.4) | 3.0 (1.6, 4.4)  1.4 (0.1, 2.8) | 5.3 (4.6, 6.0)  4.2 (3.6, 4.9) | 6.7 (6.0, 7.4)  4.8 (4.1, 5.4) | 1.4 (0.7, 2.2)  0.5 (-0.2, 1.3) |
| Self-reported adherence to public health recommendations  Low  High | 12.2 (9.5, 14.9)  10.3 (9.3, 11.2) | 13.7 (10.9, 16.5)  12.6 (11.6, 13.5) | 1.5 (-1.5, 4.5)  2.3 (1.3, 3.3) | 7.3 (5.9, 8.8)  4.4 (3.9, 4.9) | 5.6 (4.2, 7.0)  5.7 (5.2, 6.2) | -1.7 (-3.3, -0.2)  1.3 (0.7, 1.8) |
| Worried about own mental health  No  Yes | 9.8 (8.7, 10.8)  12.6 (10.9, 14.4) | 10.0 (9.0, 10.2)  20.7 (19.2, 22.2) | 0.2 (-0.8, 1.2)  8.1 (6.3, 9.9) | 4.2 (3.6, 4.7)  6.5 (5.5, 7.4) | 4.4 (3.9, 4.8)  9.5 (8.7, 10.3) | 0.2 (-0.4, 0.8)  3.1 (2.1, 4.0) |
| Worried about being unemployed  No  Yes | 10.0 (9.0, 10.9)  13.6 (11.3, 15.9) | 11.8 (10.9, 12.8)  17.5 (15.2, 19.8) | 1.9 (0.8, 2.9)  4.0 (1.5, 6.5) | 4.2 (3.7, 4.8)  7.8 (6.3, 8.9) | 5.2 (4.7, 5.7)  8.1 (6.9, 9.3) | 1.0 (0.4, 1.6)  0.5 (-0.8, 1.9) |
| Worried about not being able to pay bills  No  Yes | 10.1 (9.2, 11.1)  12.9 (15.8, 20.6) | 11.8 (10.9, 12.8)  18.2 (15.8, 20.6) | 1.7 (0.7, 2.8)  5.3 (2.6, 7.9) | 4.4 (3.8, 4.9)  7.1 (5.8, 8.3) | 5.2 (4.8, 5.7)  8.3 (7.1, 9.5) | 0.9 (0.3, 1.5)  1.2 (-0.2, 2.5) |
| Worried about not being able to visit people who depend on you  No  Yes | 9.9 (8.9, 10.9)  12.7 (10.8, 14.6) | 11.8 (10.8, 12.8)  15.9 (14.0, 17.8) | 1.9 (0.8, 3.0)  3.2 (1.2, 5.3) | 4.1 (3.6, 4.7)  6.9 (59, 7.9) | 4.9 (4.4, 5.4)  8.4 (7.5, 9.4) | 0.8 (0.2, 1.4)  1.5 (0.4, 2.6) |

^†^ Weighted for non-response

**Table S6. Weighted means and differences for change in MDI and GAD-7 scores from cycle 23 to 24, time above the median, Nicotine Dependence in Teens study, Montreal, Canada 2017-2021 (n=330)**

|  | *MDI†* | | | *GAD-7*^†^ | | |
| --- | --- | --- | --- | --- | --- | --- |
| Characteristics at cycle 24 | Mean cycle 23  (95% CI) | Mean cycle 24  (95% CI) | Mean Δ  (95% CI) | Mean cycle 23  (95% CI) | Mean cycle 24  (95% CI) | Mean Δ  (95% CI) |
| All | 10.1 (9.2, 11.0) | 12.1 (11.2, 13.1) | 2.0 (1.1, 3.0) | 4.6 (4.0, 5.1) | 5.9 (5.4, 6.4) | 1.3 (0.8, 1.9) |
| History of a mood/anxiety disorder diagnosis  No  Yes | 9.3 (7.3, 9.3)  15.2 (13.5, 16.9) | 10.7 (9.6, 11.8)  16.2 (14.4, 18.0) | 2.4 (1.3, 3.5)  1.0 (-0.9, 2.8) | 3.3 (2.7, 3.8)  8.2 (7.3, 9.1) | 5.1 (4.5, 5.6)  8.3 (7.4, 9.3) | 1.8 (1.2, 2.4)  0.1 (-0.9, 1.1) |
| Sex  Male  Female | 8.5 (7.0, 9.9)  11.1 (9.9, 12.2) | 9.7 (8.2, 11.2)  13.6 (12.4, 14.8) | 1.3 (-0.2, 2.8)  2.5 (1.3, 3.7) | 3.3 (2.4, 4.1)  5.4 (4.8, 6.1) | 4.6 (3.8, 5.4)  6.8 (6.1, 7.4) | 1.3 (0.5, 2.2)  1.4 (0.7, 2.0) |
| Ancestry  European  Other | 9.5 (8.4, 10.5)  12.5 (10.5, 14.5) | 11.8 (10.7, 13.0)  14.2 (12.0, 16.3) | 2.4 (1.3, 3.5)  1.7 (-0.4, 3.8) | 4.4 (3.8, 5.0)  5.3 (4.1, 6.4) | 5.8 (5.2, 6.4)  6.8 (5.7, 8.0) | 1.4 (0.8, 2.0)  1.6 (0.4, 2.7) |
| Born in Canada  No  Yes | 10.2 (6.4, 14.0)  10.1 (9.1, 11.0) | 11.8 (7.9, 15.8)  12.1 (11.2, 13.1) | 1.6 (-2.3, 5.5)  2.1 (1.1, 3.0) | 5.6 (3.4, 7.8)  4.5 (4.0, 5.0) | 5.5 (3.4, 7.7)  5.9 (5.4, 6.5) | 0.0 (-2.2, 2.1)  1.4 (0.9, 2.0) |
| Lives in urban/suburban setting  No  Yes | 10.2 (8.3, 12.1)  10.1 (9.0, 11.1) | 11.4 (9.4, 13.4)  12.4 (11.3, 13.5) | 1.2 (-0.7, 3.1)  2.3 (1.2, 3.4) | 4.2 (3.1, 5.2)  4.7 (4.1, 5.3) | 5.1 (4.0, 6.1)  6.2 (5.6, 6.8) | 0.9 (-0.2, 2.0)  1.5 (0.9, 2.0) |
| Lives alone  No  Yes | 10.1 (9.1, 11.0)  10.2 (7.7, 12.8) | 12.0 (11.0, 13.0)  13.1 (10.4, 15.8) | 1.9 (0.9, 2.9)  2.9 (0.3, 5.5) | 4.5 (4.0, 5.1)  4.9 (3.4, 6.4) | 5.9 (4.0, 5.1)  6.1 (4.6, 7.6) | 1.4 (0.8, 1.9)  1.2 (-0.3, 2.7) |
| Lives with children  No  Yes | 10.5 (9.2, 11.8)  9.7 (8.4, 11.0) | 12.6 (11.2, 14.0)  11.7 (10.4, 13.0) | 2.1 (0.8, 3.5)  2.0 (0.7, 3.3) | 4.8 (4.0, 5.5)  4.4 (3.7, 5.1) | 6.1 (5.4, 6.9)  5.7 (5.0, 6.4) | 1.4 (0.6, 2.1)  1.3 (0.6, 2.0) |
| Single parent in a household with children  No  Yes | 9.3 (7.9, 10.6)  15.3 (10.5, 20.1) | 11.2 (9.9, 12.6)  17.2 (12.3, 22.1) | 2.0 (0.6, 3.4)  1.9 (-3.2, 7.1) | 4.2 (3.4, 5.0)  6.1 (3.4, 8.8) | 5.6 (4.9, 6.3)  6.8 (4.4, 9.2) | 1.4 (0.6, 2.2)  0.7 (-2.0, 3.3) |
| Age of children in households with children  ≥ 1 child age ≤ 5 years  Children all age > 5 years | 9.1 (7.7, 10.4)  15.1 (11.3, 18.9) | 11.2 (9.8, 12.6)  15.7 (11.8, 19.6) | 2.1 (0.7, 3.6)  0.6 (-3.5, 4.8) | 3.8 (3.0, 4.6)  8.6 (6.5, 10.6) | 5.6 (4.9, 6.4)  6.2 (4.2, 8.2) | 1.8 (1.0, 2.6)  -2.4 (-4.4, -0.3) |
| Highest education  More than high school  High school | 10.1 (9.1, 11.0)  11.4 (7.0, 15.8) | 12.1 (11.1, 13.1)  13.4 (8.8, 17.9) | 2.0 (1.1, 3.0)  2.0 (-2.5, 6.4) | 4.4 (3.9, 5.0)  6.7 (4.4, 9.0) | 5.9 (5.3, 6.4)  5.8 (3.5, 8.0) | 1.4 (0.9, 2.0)  -0.9 (3.2, 1.3) |
| Unemployed  No  Yes | 9.7 (8.6, 10.7)  11.8 (9.8, 13.9) | 11.7 (10.7, 12.8)  13.7 (11.5, 15.9) | 2.1 (1.1, 3.1)  1.9 (-0.3, 4.0) | 4.5 (3.9, 5.1)  4.9 (3.7, 6.1) | 5.7 (5.2, 6.3)  6.7 (5.5, 7.9) | 1.3 (0.7, 1.8)  1.8 (0.6, 3.0) |
| Household income  < $50,000  ≥ $50,000 | 13.9 (12.0, 15.8)  9.0 (8.0, 10.0) | 13.2 (11.2, 15.3)  11.8 (10.8, 12.9) | -0.7 (-2.7, 1.3)  2.8 (1.8, 3.8) | 6.4 (5.3, 7.6)  4.1 (3.5, 4.7) | 6.1 (5.0, 7.2)  5.9 (5.3, 6.5) | -0.3 (-1.4, 0.8)  1.8 (1.2, 2.4) |
| Work-at-home status during COVID-19 among employed  Works at home  Works outside home/mixed | 11.3 (9.9, 12.7)  9.2 (7.9, 10.4) | 13.5 (12.1, 15.0)  11.0 (9.8, 12.3) | 2.3 (0.8, 3.7)  1.9 (0.7, 3.1) | 5.1 (4.3, 5.8)  4.2 (3.5, 4.9) | 6.6 (5.9, 7.4)  5.4 (4.7, 6.0) | 1.6 (0.8, 2.4)  1.2 (0.5, 1.9) |
| Self-reported adherence to public health recommendations  Low  High | 12.6 (10.2, 15.0)  9.6 (8.7, 10.6) | 14.1 (11.6, 16.6)  11.8 (10.8, 12.8) | 1.5 (-1.0, 3.9)  2.1 (1.1, 3.1) | 7.4 (6.0, 8.7)  4.1 (3.6, 4.6) | 6.7 (5.3, 8.0)  5.8 (5.2, 6.3) | -0.7 (-2.1, 0.6)  1.7 (1.1, 2.2) |
| Worried about own mental health  No  Yes | 9.1 (8.2, 10.1)  13.9 (11.9, 15.9) | 9.4 (8.6, 10.2)  23.2 (21.5, 24.8) | 0.3 (-0.7, 1.2)  9.3 (7.4, 11.2) | 3.8 (3.2, 4.3)  7.7 (6.6, 8.8) | 4.4 (3.9, 4.8)  12.1 (11.2, 12.9) | 0.6 (0.1, 1.2)  4.4 (3.3, 5.5) |
| Worried about being unemployed  No  Yes | 9.5 (8.6, 10.5)  13.8 (11.2, 16.3) | 11.5 (10.5, 12.5)  16.3 (13.7, 19.0) | 2.0 (1.0, 3.0)  2.5 (-0.1, 5.2) | 4.3 (3.8, 4.9)  6.1 (4.7, 7.6) | 5.6 (5.1, 6.2)  7.9 (6.5, 9.4) | 1.3 (0.7, 1.8)  1.8 (0.3, 3.3) |
| Worried about not being able to pay bills  No  Yes | 9.1 (8.1, 10.0)  16.6 (14.3, 19.0) | 11.3 (10.3, 12.3)  17.2 (14.7, 19.7) | 2.3 (1.3, 3.3)  0.6 (-1.9, 3.1) | 4.1 (3.6, 4.7)  7.2 (5.8, 8.6) | 5.6 (5.1, 6.1)  7.9 (6.5, 9.2) | 1.5 (0.9, 2.0)  0.7 (-0.7, 2.1) |
| Worried about not being able to visit people who depend on you  No  Yes | 9.4 (8.4, 10.4)  12.3 (10.4, 14.2) | 11.3 (10.2, 12.4)  14.9 (12.9, 16.8) | 1.9 (0.8, 3.0)  2.6 (0.6, 4.5) | 4.1 (3.5, 4.7)  6.2 (5.1, 7.2) | 5.5 (5.0, 6.1)  7.1 (6.0, 8.2) | 1.5 (0.9, 2.1)  0.9 (-0.1, 2.0) |

^†^ Weighted for non-response

**Table S7. Unweighted means and differences) for change in MDI and GAD-7 scores from cycle 23 to 24, time below the median, Nicotine Dependence in Teens study, Montreal, Canada 2017-2021 (n=343)**

|  | *MDI†* | | | *GAD-7*^†^ | | |
| --- | --- | --- | --- | --- | --- | --- |
| Characteristics at cycle 24 | Mean cycle 23  (95% CI) | Mean cycle 24  (95% CI) | Mean Δ  (95% CI) | Mean cycle 23  (95% CI) | Mean cycle 24  (95% CI) | Mean Δ  (95% CI) |
| All | 10.2 (9.4, 11.1) | 12.3 (11.4, 13.3) | 2.1 (1.2, 3.0) | 4.6 (4.1, 5.1) | 5.6 (5.1, 6.1) | 1.0 (0.5, 1.5) |
| History of a mood/anxiety disorder diagnosis  No  Yes | 9.2 (8.2, 10.1)  14.0 (12.9, 15.7) | 11.2 (10.2, 12.2)  16.5 (14.6, 18.3) | 2.0 (1.0, 3.0)  2.5 (0.6, 4.4) | 3.9 (3.4, 4.5)  6.9 (5.9, 7.8) | 5.1 (4.6, 5.6)  7.2 (6.3, 8.2) | 1.2 (0.6, 1.7)  0.4 (-0.7, 1.4) |
| Sex  Male  Female | 9.1 (7.9, 10.4)  11.1 (10.0, 12.2) | 11.2 (9.9, 12.6)  13.2 (12.0, 14.4) | 2.1 (0.7, 3.5)  2.1 (0.9, 3.4) | 3.7 (3.0, 4.4)  5.3 (4.7, 6.0) | 4.6 (3.9, 5.3)  6.4 (5.8, 7.0) | 0.9 (0.2, 1.6)  1.1 (0.4, 1.7) |
| Ancestry  European  Other | 10.1 (9.1, 11.1)  10.8 (9.0, 12.6) | 11.9 (10.9, 13.0)  13.8 (11.9, 15.6) | 1.8 (0.8, 2.9)  3.0 (1.1, 5.0) | 4.6 (4.1, 5.2)  4.6 (3.6, 5.6) | 5.6 (5.0, 6.2)  6.0 (5.1, 6.9) | 0.8 (0.3, 1.4)  1.3 (0.2, 2.3) |
| Born in Canada  No  Yes | 11.4 (8.2, 14.6)  10.1 (9.3, 11.0) | 11.7 (8.2, 15.1)  12.4 (11.5, 13.3) | 0.3 (-3.2, 3.7)  2.3 (1.3, 3.2) | 3.6 (1.8, 5.4)  4.7 (4.2, 5.3) | 4.9 (3.0, 6.7)  5.7 (5.2, 6.2) | 1.3 (-0.6, 3.2)  1.0 (0.5, 1.5) |
| Lives in urban/suburban setting  No  Yes | 11.9 (9.6, 14.3)  10.0 (9.1, 10.9) | 13.4 (10.9, 16.0)  12.2 (11.2, 13.2) | 1.5 (-1.1, 4.1)  2.2 (1.3, 3.2) | 5.2 (3.9, 6.5)  4.5 (4.0, 5.1) | 6.1 (4.7, 7.4)  5.5 (5.0, 6.0) | 0.8 (-0.5, 2.2)  1.0 (0.4, 1.5) |
| Lives alone  No  Yes | 10.1 (9.1, 11.0)  10.9 (8.9, 13.0) | 12.0 (11.0, 13.0)  14.4 (12.2, 16.6) | 1.9 (0.9, 2.9)  3.5 (1.2, 5.7) | 4.5 (4.0, 5.1)  5.1 (3.9, 6.3) | 5.4 (4.9, 5.9)  6.4 (5.2, 7.6) | 0.9 (0.3, 1.4)  1.3 (0.1, 2.5) |
| Lives with children  No  Yes | 10.6 (9.5, 11.7)  9.6 (8.2, 11.0) | 13.2 (12.0, 14.3)  11.0 (9.5, 12.5) | 2.6 (1.4, 3.7)  1.4 (-0.1, 2.9) | 4.8 (4.2, 5.4)  4.4 (3.6, 5.2) | 5.8 (5.1, 6.4)  5.3 (4.5, 6.1) | 1.0 (0.4, 1.6)  0.9 (0.1, 1.7) |
| Single parent in a household with children  No  Yes | 9.5 (8.1, 10.9)  10.8 (5.7, 15.8) | 11.1 (9.6, 12.5)  10.2 (5.0, 15.4) | 1.6 (0, 3.2)  -0.6 (6.2, 5.1) | 4.3 (3.5, 5.2)  4.8 (1.9, 7.7) | 5.4 (4.7, 6.2)  3.3 (0.7, 6.0) | 1.1 (0.3, 1.9)  -1.4 (-4.2, 1.4) |
| Age of children in households with children  ≥ 1 child age ≤ 5 years  Children all age > 5 years | 9.5 (8.1, 11.0)  10.0 (6.1, 13.9) | 10.7 (9.2, 12.2)  13.0 (9.0, 17.0) | 1.2 (-0.5, 2.8)  3.0 (-1.4, 7.4) | 4.4 (3.6, 5.2)  4.1 (1.8, 6.5) | 5.3 (4.5, 6.0)  5.4 (3.2, 7.5) | 0.9 (0.0, 1.7)  1.2 (-1.1, 3.5) |
| Highest education  More than high school  High school | 10.3 (9.4, 11.2)  10.4 (3.4, 17.4) | 12.5 (11.5, 13.4)  12.6 (5.1, 20.1) | 2.2 (1.2, 3.2)  2.2 (-5.4, 9.8) | 4.7 (4.2, 5.2)  5.0 (1.4, 8.6) | 5.6 (5.1, 6.1)  5.7 (2.1, 9.3) | 0.9 (0.4, 1.4)  0.7 (-3.0, 4.4) |
| Unemployed  No  Yes | 10.2 (9.3, 11.1)  10.3 (7.9, 12.6) | 12.0 (11.0, 12.9)  15.0 (12.5, 17.5) | 1.7 (0.8, 2.7)  4.7 (2.2, 7.2) | 4.5 (4.0, 5.0)  5.5 (4.2, 6.7) | 5.4 (4.9, 5.9)  6.7 (5.5, 8.0) | 0.9 (0.4, 1.4)  1.3 (-0.1, 2.6) |
| Household income  < $50,000  ≥ $50,000 | 11.5 (9.7, 13.3)  9.9 (8.9, 10.8) | 14.0 (12.1, 16.0)  11.9 (10.9, 12.9) | 2.6 (0.6, 4.5)  2.0 (1.0, 3.1) | 4.9 (3.9, 5.9)  4.6 (4.0, 5.1) | 5.6 (4.6, 6.6)  5.6 (5.0, 6.1) | 0.7 (-0.4, 1.8)  1.0 (0.5, 1.6) |
| Work-at-home status during COVID-19 among employed  Works at home  Works outside home/mixed | 10.6 (9.3, 11.8)  9.9 (8.8, 11.1) | 13.8 (12.5, 15.1)  11.0 (9.8, 12.3) | 3.3 (1.9, 4.6)  1.1 (-0.2, 2.3) | 4.8 (4.1, 5.5)  4.5 (3.8, 5.1) | 6.3 (5.6, 7.0)  5.0 (4.3, 5.6) | 1.5 (0.8, 2.2)  0.5 (-0.2, 1.2) |
| Self-reported adherence to public health recommendations  Low  High | 11.4 (8.6, 14.2)  10.1 (9.2, 11.0) | 12.9 (9.9, 15.9)  12.3 (11.3, 13.2) | 1.5 (-1.6, 4.5)  2.2 (1.2, 3.1) | 6.2 (4.7, 7.7)  4.5 (4.0, 5.0) | 5.3 (3.7, 6.8)  5.6 (5.1, 6.1) | -0.9 (-2.5, 0.7)  1.1 (0.6, 1.7) |
| Worried about own mental health  No  Yes | 9.2 (8.2, 10.1)  13.5 (11.9, 15.2) | 9.6 (8.8, 10.5)  20.9 (19.4, 22.5) | 0.4 (-0.6, 1.4)  7.4 (5.7, 9.2) | 3.8 (3.3, 4.4)  7.1 (6.1, 8.0) | 4.0 (3.6, 4.5)  10.3 (9.6, 11.1) | 0.2 (-0.3, 0.7)  3.3 (2.3, 4.2) |
| Worried about being unemployed  No  Yes | 9.6 (8.7, 10.5)  14.1 (11.9, 16.4) | 11.4 (10.5, 12.3)  18.2 (15.9, 20.6) | 1.8 (0.8, 2.8)  4.1 (1.6, 6.5) | 4.2 (3.7, 4.7)  7.0 (5.8, 8.3) | 5.1 (4.6, 5.6)  8.2 (7.0, 9.4) | 0.9 (0.4, 1.5)  1.2 (-0.2, 2.5) |
| Worried about not being able to pay bills  No  Yes | 9.7 (8.8, 10.6)  14.2 (11.8, 16.6) | 11.4 (10.5, 12.4)  19.1 (16.5, 21.6) | 1.7 (0.8, 2.7)  4.9 (2.2, 7.5) | 4.2 (3.7, 4.7)  7.3 (6.0, 8.6) | 5.0 (4.6, 5.5)  9.3 (8.0, 10.6) | 0.8 (0.3, 1.3)  2.0 (0.6, 3.4) |
| Worried about not being able to visit people who depend on you  No  Yes | 9.3 (8.3, 10.2)  13.6 (11.9, 15.3) | 11.5 (10.5, 12.5)  15.2 (13.3, 17.1) | 2.3 (1.2, 3.3)  1.6 (-0.3, 35) | 3.9 (3.4, 5.3)  7.2 (6.2, 8.1) | 4.8 (4.3, 5.3)  8.1 (7.2, 9.1) | 1.0 (0.4, 1.5)  0.9 (-0.1, 2.0) |

^†^ Unweighted for non-response

**Table S8. Unweighted means and differences) for change in MDI and GAD-7 scores from cycle 23 to 24, time above the median, Nicotine Dependence in Teens study, Montreal, Canada 2017-2021 (n=330)**

|  | *MDI†* | | | *GAD-7*^†^ | | |
| --- | --- | --- | --- | --- | --- | --- |
| Characteristics at cycle 24 | Mean cycle 23  (95% CI) | Mean cycle 24  (95% CI) | Mean Δ  (95% CI) | Mean cycle 23  (95% CI) | Mean cycle 24  (95% CI) | Mean Δ  (95% CI) |
| All | 10.3 (9.4, 11.2) | 12.3 (11.3, 13.3) | 2.0 (1.0, 3.0) | 4.7 (4.2, 5.2) | 6.0 (5.5, 6.5) | 1.3 (0.8, 1.8) |
| History of a mood/anxiety disorder diagnosis  No  Yes | 8.5 (7.5, 9.5)  15.0 (13.4, 16.7) | 10.7 (9.6, 11.8)  16.5 (14.7, 18.4) | 2.2 (1.1, 3.3)  1.5 (-0.3, 3.4) | 3.6 (3.1, 4.2)  7.6 (6.7, 5.5) | 5.1 (4.5, 5.7)  8.4 (7.4, 9.4) | 1.5 (0.9, 2.1)  0.8 (-0.2, 1.8) |
| Sex  Male  Female | 9.5 (8.0, 10.9)  10.8 (9.6, 12.0) | 10.6 (9.0, 12.2)  13.4 (12.1, 14.6) | 1.1 (-0.4, 2.7)  2.6 (1.3, 3.8) | 3.9 (3.1, 4.7)  5.2 (4.6, 5.9) | 5.1 (4.3, 5.9)  6.6 (5.9, 7.3) | 1.2 (0.3, 2.0)  1.4 (0.7, 2.0) |
| Ancestry  European  Other | 9.8 (8.7, 10.8)  11.9 (9.9, 13.9) | 12.1 (10.9, 13.2)  14.0 (11.8, 16.2) | 2.3 (1.1, 3.4)  2.1 (-0.1, 4.3) | 4.5 (3.9, 5.1)  5.1 (4.0, 6.3) | 5.7 (5.1, 6.3)  7.1 (5.9, 8.2) | 1.2 (0.6, 1.8)  1.9 (0.7, 3.1) |
| Born in Canada  No  Yes | 9.9 (5.7, 14.2)  10.1 (9.4, 11.2) | 14.5 (9.9, 19.0)  12.2 (11.2, 13.2) | 4.5 (0.0, 9.0)  1.9 (0.9, 2.9) | 6.0 (3.6, 8.4)  4.6 (4.1, 5.2) | 7.4 (4.8, 9.9)  5.9 (5.4, 6.5) | 1.4 (-1.2, 3.9)  1.3 (0.8, 1.8) |
| Lives in urban/suburban setting  No  Yes | 10.5 (8.5, 12.5)  10.3 (9.2, 11.3) | 12.3 (10.1, 14.5)  12.3 (11.2, 13.4) | 1.8 (-0.3, 4.0)  2.1 (1.0, 3.2) | 4.6 (3.5, 5.7)  4.8 (4.2, 5.3) | 5.8 (4.7, 7.0)  6.0 (5.4, 6.7) | 1.2 (0.1, 2.4)  1.3 (0.7, 1.9) |
| Lives alone  No  Yes | 10.1 (9.2, 11.1)  11.2 (8.7, 13.8) | 12.1 (11.1, 13.2)  13.1 (10.3, 15.8) | 2.0 (1.0, 3.1)  1.8 (-0.9, 4.6) | 4.6 (4.1, 5.2)  5.1 (5.4, 6.5) | 5.9 (5.4, 6.5)  6.4 (4.9, 8.0) | 1.3 (0.7, 1.9)  1.3 (-0.3, 2.8) |
| Lives with children  No  Yes | 10.9 (9.6, 12.2)  9.6 (8.4, 10.9) | 12.2 (10.8, 13.6)  12.4 (11.0, 13.8) | 1.3 (-0.1, 2.6)  2.8 (1.4, 4.1) | 4.9 (4.2, 5.6)  4.5 (3.8, 5.2) | 5.8 (5.0, 6.6)  6.2 (5.4, 6.9) | 0.9 (0.1, 1.6)  1.7 (1.0, 2.4) |
| Single parent in a household with children  No  Yes | 9.4 (8.1, 10.7)  11.8 (7.7, 15.9) | 11.8 (10.4, 13.3)  17.7 (13.0, 22.4) | 2.4 (0.9, 4.0)  5.9 (1.1, 10.7) | 4.4 (3.6, 5.2)  5.2 (2.8, 7.6) | 6.0 (5.2, 6.8)  7.9 (5.5, 10.4) | 1.6 (0.8, 2.4)  2.7 (0.3, 5.2) |
| Age of children in households with children  ≥ 1 child age ≤ 5 years  Children all age > 5 years | 9.5 (8.2, 10.9)  11.1 (7.5, 14.7) | 12.0 (10.5, 13.5)  15.8 (11.6, 20.1) | 2.5 (0.9, 4.1)  4.7 (0.5, 9.0) | 4.3 (3.5, 5.0)  6.5 (4.4, 8.7) | 6.1 (5.3, 7.0)  6.6 (4.4, 8.9) | 1.9 (1.1, 2.7)  0.1 (-2.1, 2.3) |
| Highest education  More than high school  High school | 10.3 (9.3, 11.2)  13.0 (8.4, 17.6) | 12.2 (11.2, 13.3)  16.2 (11.4, 21.1) | 2.0 (1.0, 3.0)  3.2 (-1.6, 8.1) | 4.6 (4.1, 5.1)  7.1 (4.7, 9.6) | 5.9 (5.4, 6.5)  8.1 (5.6, 10.6) | 1.3 (0.8, 1.8)  0.9 (-1.6, 3.5) |
| Unemployed  No  Yes | 9.8 (8.8, 10.8)  12.2 (10.2, 14.3) | 11.8 (10.8, 13.0)  13.9 (11.8, 16.2) | 2.1 (1.0, 3.2)  1.7 (-0.5, 3.9) | 4.5 (3.9, 5.0)  5.7 (4.6, 6.8) | 5.7 (5.1, 6.3)  7.3 (6.1, 8.5) | 1.2 (0.6, 1.8)  1.6 (0.4, 2.8) |
| Household income  < $50,000  ≥ $50,000 | 13.5 (11.4, 15.5)  9.5 (8.5, 10.5) | 13.3 (11.1, 15.5)  12.1 (11.0, 13.2) | -0.1 (-2.3, 2.0)  2.6 (1.5, 3.7) | 6.2 (5.0, 7.3)  4.4 (3.8, 4.9) | 6.5 (5.3, 7.7)  5.9 (5.3, 6.5) | 0.3 (-0.9, 1.5)  1.5 (0.9, 2.1) |
| Work-at-home status during COVID-19 among employed  Works at home  Works outside home/mixed | 11.2 (9.8, 12.6)  9.5 (8.3, 10.8) | 13.6 (12.1, 15.0)  11.3 (10.0, 12.6) | 2.4 (0.9, 3.8)  1.7 (0.4, 3.1) | 5.1 (4.3, 5.8)  4.4 (3.7, 5.1) | 6.4 (5.7, 7.2)  5.6 (4.9, 6.3) | 1.4 (0.6, 2.2)  1.2 (0.5, 1.9) |
| Self-reported adherence to public health recommendations  Low  High | 11.3 (8.8, 13.8)  10.1 (9.2, 11.1) | 13.7 (11.0, 16.4)  12.1 (11.0, 13.1) | 2.5 (-0.2, 5.1)  1.9 (0.9, 3.0) | 5.9 (4.5, 7.3)  4.5 (4.0, 5.1) | 6.2 (4.8, 7.7)  6.0 (5.4, 6.5) | 0.3 (-1.1, 1.8)  1.4 (0.9, 2.0) |
| Worried about own mental health  No  Yes | 9.4 (8.4, 10.4)  14.1 (12.1, 16.2) | 9.5 (8.7, 10.4)  24.2 (22.4, 25.9) | 0.2 (-0.8, 1.1)  10.1 (8.0, 12.1) | 4.1 (3.5, 4.6)  7.4 (6.3, 8.5) | 4.6 (4.1, 5.1)  11.9 (10.9, 12.9) | 0.5 (0.0, 1.1)  4.5 (3.4, 5.7) |
| Worried about being unemployed  No  Yes | 9.7 (8.8, 10.7)  13.9 (11.4, 16.3) | 11.7 (10.6, 12.7)  16.3 (13.7, 18.9) | 2.0 (0.9, 3.0)  2.4 (-0.2, 5.1) | 4.4 (3.9, 4.9)  6.7 (5.3, 8.0) | 5.6 (5.1, 6.2)  8.3 (6.9, 9.7) | 1.2 (0.7, 1.8)  1.6 (0.2, 3.1) |
| Worried about not being able to pay bills  No  Yes | 9.3 (8.3, 10.2)  16.1 (14.5, 19.5) | 11.5 (10.4, 12.5)  17.0 (14.5, 19.5) | 2.2 (1.2, 3.3)  0.9 (-1.6, 3.4) | 4.3 (3.7, 4.8)  7.1 (5.8, 8.4) | 5.6 (5.0, 6.2)  8.2 (6.8, 9.5) | 1.3 (0.8, 1.9)  1.1 (-0.3, 2.5) |
| Worried about not being able to visit people who depend on you  No  Yes | 9.6 (8.6, 10.6)  12.3 (10.5, 14.1) | 11.5 (10.4, 12.7)  14.4 (12.5, 16.4) | 2.0 (0.8, 3.1)  2.2 (0.2, 4.1) | 4.2 (3.6, 4.8)  6.2 (5.2, 7.2) | 5.6 (5.0, 6.2)  7.0 (6.0, 8.1) | 1.5 (0.8, 2.1)  0.8 (-0.2, 1.9) |

^†^ Unweighted for non-response

**Table S9. Unweighted mean of individual differences (Δ) and standardized mean changes (SMΔ) for MDI and**

**GAD-7 scores from cycle 23 to 24, Nicotine Dependence in Teens study, Montreal, Canada 2017-2021, n=673**

|  | *MDI* | |  | *GAD-7* | |
| --- | --- | --- | --- | --- | --- |
| Characteristics | Mean Δ^‡^  (95% CI) | SMΔ^*^  (95% CI) |  | Mean Δ^‡^  (95% CI) | SMΔ^*^  (95% CI) |
| All | 2.1 (1.4, 2.7) | **0.24 (0.16, 0.31)** |  | 2.1 (1.4, 2.7) | **0.24 (0.16, 0.31)** |
| History of a mood/anxiety disorder diagnosis  No  Yes | 2.1 (1.3, 2.9)  2.0 (0.6, 3.3) | -0.01 (-0.23, 0.21) |  | 2.1 (1.3, 2.9)  2.0 (0.6, 3.3) | -0.01 (-0.23, 0.21) |
| Sex  Male  Female | 1.7 (0.6, 2.7)  2.4 (1.5, 3.2) | 0.08 (-0.07, 0.24) |  | 1.7 (0.6, 2.7)  2.4 (1.5, 3.2) | 0.08 (-0.07, 0.24) |
| Ancestry  European  Other | 2.1 (1.3, 2.8)  2.6 (1.1, 4.0) | 0.06 (-0.14, 0.25) |  | 2.1 (1.3, 2.8)  2.6 (1.1, 4.0) | 0.06 (-0.14, 0.25) |
| Born in Canada  No  Yes | 2.0 (-0.8, 4.7)  2.1 (1.4, 2.8) | 0.01 (-0.36, 0.40) |  | 2.0 (-0.8, 4.7)  2.1 (1.4, 2.8) | 0.01 (-0.36, 0.40) |
| Lives in urban/suburban setting  No  Yes | 1.7 (0.1, 3.3)  2.2 (1.4, 2.9) | 0.05 (-0.19, 0.30) |  | 1.7 (0.1, 3.3)  2.2 (1.4, 2.9) | 0.05 (-0.19, 0.30) |
| Lives alone  No  Yes | 2.0 (1.2, 2.7)  2.8 (1.0, 4.5) | 0.10 (-0.10, 0.31) |  | 2.0 (1.2, 2.7)  2.8 (1.0, 4.5) | 0.10 (-0.10, 0.31) |
| Lives with children  No  Yes | 2.0 (1.1, 2.9)  2.2 (1.2, 3.2) | 0.02 (-0.14, 0.18) |  | 2.0 (1.1, 2.9)  2.2 (1.2, 3.2) | 0.02 (-0.14, 0.18) |
| Single parent in a household with children  No  Yes | 2.1 (1.0, 3.2)  3.5 (-0.2, 7.2) | 0.17 (-0.48, 0.82) |  | 2.1 (1.0, 3.2)  3.5 (-0.2, 7.2) | 0.17 (-0.48, 0.82) |
| Age of children in households with children  ≥ 1 child age ≤ 5 years  Children all age > 5 years | 1.9 (0.8, 3.1)  4.0 (0.9, 7.1) | 0.24 (-0.23, 0.76) |  | 1.9 (0.8, 3.1)  4.0 (0.9, 7.1) | 0.24 (-0.23, 0.76) |
| Highest education  More than high school  High school | 2.1 (1.4, 2.8)  2.9 (-1.1, 7.0) | 0.10 (-0.26, 0.71) |  | 2.1 (1.4, 2.8)  2.9 (-1.1, 7.0) | 0.10 (-0.26, 0.71) |
| Unemployed  No  Yes | 1.9 (1.2, 26)  3.0 (1.3, 4.6) | 0.12 (-0.11, 0.40) |  | 1.9 (1.2, 26)  3.0 (1.3, 4.6) | 0.12 (-0.11, 0.40) |
| Household income  < $50,000  ≥ $50,000 | 1.3 (-0.2, 2.8)  2.3 (1.5, 3.1) | 0.12 (-0.08, 0.32) |  | 1.3 (-0.2, 2.8)  2.3 (1.5, 3.1) | 0.12 (-0.08, 0.32) |
| Work-at-home status during COVID-19 among employed  Works at home  Works outside home/mixed | 2.8 (1.6, 3.9)  1.4 (0.5, 2.3) | -0.17 (-0.35, 0.02) |  | 2.8 (1.6, 3.9)  1.4 (0.5, 2.3) | -0.17 (-0.35, 0.02) |
| Self-reported adherence to public health recommendations  Low  High | 2.1 (0.1, 4.1)  2.1 (1.4, 2.8) | 0 (-0.30, 0.31) |  | 2.1 (0.1, 4.1)  2.1 (1.4, 2.8) | 0 (-0.30, 0.31) |
| Worried about being unemployed  No  Yes | 1.9 (1.2, 2.6)  3.3 (1.5, 5.1) | 0.17 (-0.08, 0.43) |  | 1.9 (1.2, 2.6)  3.3 (1.5, 5.1) | 0.17 (-0.08, 0.43) |
| Worried about not being able to pay bills  No  Yes | 2.0 (1.3, 2.7)  2.7 (0.8, 4.5) | 0.08 (-0.21, 0.37) |  | 2.0 (1.3, 2.7)  2.7 (0.8, 4.5) | 0.08 (-0.21, 0.37) |
| Worried about not being able to visit people who depend on you  No  Yes | 2.1 (1.4, 2.9)  1.9 (0.5, 3.3) | -0.03 (-0.23, 0.18) |  | 2.1 (1.4, 2.9)  1.9 (0.5, 3.3) | -0.03 (-0.23, 0.18) |

^‡^Represents individual differences between MDI or GAD-7 scores in cycle 24 and 23.

*Represents differences in mean change between groups, divided by the within-group standard deviation averaged across cycle 23 and 24. 95% CIs were calculated using bootstrap resampling.

SMD with 95% CI that excluded the null are in **bold** font.
